# Reply to Schramm, L. Comment on “Li et al. *BDP1* Variants I1264M and V1347M Significantly Associated with Clinical Outcomes of Pediatric Neuroblastoma Patients Imply a New Prognostic Biomarker: A 121-Patient Cancer Genome Study. *Diagnostics* 2021, *11*, 2364”

**DOI:** 10.3390/diagnostics12030617

**Published:** 2022-03-02

**Authors:** Xiaoqing Li, Lan Sun, Andres Stucky, Lingli Tu, Jin Cai, Xuelian Chen, Zhongjun Wu, Xuhong Jiang, Shengwen Calvin Li

**Affiliations:** 1 Department of Oncology, The People’s Hospital of Bishan District, Chongqing 402760, China; 2018020067@stu.cqmu.edu.cn (X.L.); ls_000@usc.edu (L.S.); 2 Department of Hepatobiliary Surgery, The First Affiliated Hospital of Chongqing Medical University, Chongqing 400042, China; 3 Department of Otolaryngology, Keck School of Medicine, University of Southern California, Los Angeles, CA 90033, USA; astucky@usc.edu (A.S.); tulingli425@gmail.com (L.T.); xuelianc@usc.edu (X.C.); 4 Department of Oral and Maxillofacial Surgery, Zhuhai People’s Hospital, Zhuhai Hospital Affiliated with Jinan University, Zhuhai 519000, China; caijin70@163.com; 5 Department of Health Management, Zhuhai People’s Hospital, Zhuhai Hospital Affiliated with Jinan University, Zhuhai 519000, China; 6 Neuro-Oncology and Stem Cell Research Laboratory, Center for Neuroscience Research, CHOC Children’s Research Institute, Children’s Hospital of Orange County (CHOC), 1201 West La Veta Ave., Orange, CA 92868-3874, USA; shengwel@uci.edu; 7 Department of Neurology, Irvine School of Medicine, University of California, 200 S Manchester Ave. Ste. 206, Orange, CA 92868, USA

We thank Professor Laura Schramm for her comment on the history and clarification of BDP1 nomenclature, her contribution to gene cloning [1], and functional characterizations [2]. We apologize for the oversight [3] and confusion on the misquotation [4]. We appreciated her feedback.

Specifically, Professor Laura Schramm pointed out that our article miscited Gensler’s study entitled “Negative Regulation of HER2 Signaling by the PEST-type Protein-tyrosine Phosphatase BDP1” as evidence for the TFIIIIB-associated BDP1 subunit playing a pivotal role in breast cancer. She went through the history and clarification of BDP1 nomenclature. She correctly pointed out the difference between the PEST-motif containing Protein-tyrosine Phosphatase BDP1, aka, brain-derived phosphatase 1 (BDP1), and the TFIIIIB-associated BDP1 subunit, aka., B Double Prime 1 [3].

In the introduction of our article, we stated: “Along the line, we focus on the human gene BDP1 (B Double Prime 1), located on chromosome 5q13, encodes a subunit of RNA Polymerase III Transcription Initiation Factor IIIB (TFIIIB) [5], which is suppressed by BRCA1 [6]. However, neither the BDP1’s physiology nor its pathology in humans is fully known, unlike Drosophila melanogaster [7,8]. The manuscript attempted to energize the novelty of BDP1 in neuroblastoma, even though the concept of BDP1’s cancer-involved is emerging with colorectal cancers [9,10], lung cancer [11], and breast cancer [12]. To date, the importance of BDP1 mutations in N.B. has remained relatively unstudied. Thus, we conducted this study to explore the value of BDP1 mutations in N.B. by RNA-seq, yielding a new perspective on a pediatric brain tumor neuroblastoma”.

Given the above background, Professor Laura Schramm’s comment corrected our misquotation of breast cancer [12]. Gensler’s group worked on the PEST-type Protein-tyrosine Phosphatase BDP1, aka brain-derived phosphatase 1 (BDP1), initially [12] and was renamed to PTPN18 HGNC data (Refer to Gene symbol report | HUGO Gene Nomenclature Committee (genenames.org, accessed on 18 January 2022) [3,4]). To offer a clarification, we have drawn a schematic diagram (Figure 1) to illustrate the timeline of nomenclature and the difference between PTPN18 and BDP1 and their perspectives in cancer involvement, as per Professor Schramm’s suggestion. We noticed that there is functional overlap of both PTPN18 and BDP1 in colorectal cancer [9,13]. Uniquely, silencing of PTPN18 induced ferroptosis in endometrial cancer cells [14] through p-P38-mediated GPX4/xCT downregulation [15], while downregulation of PTPN18 can inhibit proliferation and metastasis and promote apoptosis of endometrial cancer [16]. All of these genes are yet to be elucidated for their therapeutic impacts in clinal studies.

## Figures and Tables

**Figure 1:**
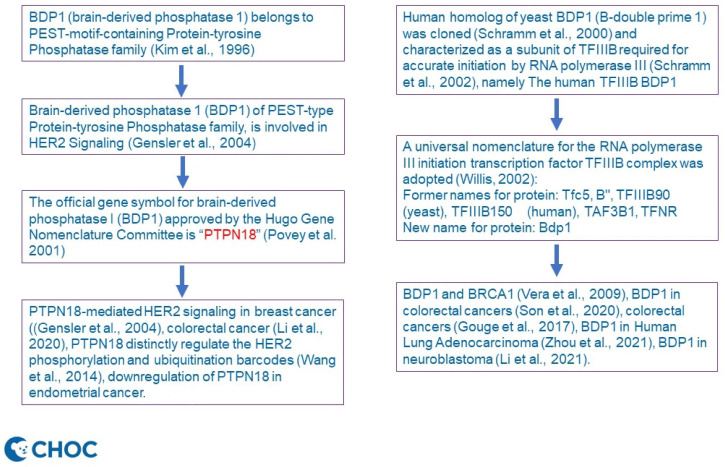
Illustrating the timeline of nomenclature and difference between PTPN18 and BDP1 and their perspective involvement in cancer (refer to citations References [1,2,5,6,7,8,9,10,11,12,13,14,15,16] for details).
